# Low Pass-rate in postgraduate surgical examination in Nigeria and its contribution to the low surgeon workforce in the country; a review article

**DOI:** 10.1051/sicotj/2018008

**Published:** 2018-09-03

**Authors:** Jonathan L. Ajah

**Affiliations:** Specialty Registrar in Surgery, Jos University Teaching Hospital, Jos Nigeria

**Keywords:** Post graduate surgical examination, Examination pass rate, Workforce and surgeon.

## Abstract

Surgical postgraduate examiners and examinees in Nigeria complain of the low pass rate at all levels of the postgraduate surgical training examinations to which several factors are contributing. For several years there has been being a persistently low surgeon workforce in the country despite having two surgeon producing institutions been for at least 37 years. A review of the probable causes was carried out to shed more light on the matter. At the time of writing there are 52 National Postgraduate Medical College of Nigeria (NPMCN) and 46 West African College of Surgeons (WACS) accredited post graduate surgery training programs in Nigeria compared with 99 in the United Kingdom (UK) and 1056 in the United States (US). Based on available data Nigeria has approximately 572 surgery residency training slots yearly compared with approximately 646 in the UK and 4225 in the US. Examination pass rate was less than 40% for primary WACS compared with 98% pass rate in USMLE (United States Medical Licensing Examination) 3, pass rate at part I was 28.8% for WACS compared with 37% at MRCS (Membership Royal College of Surgeons) part A and 57% for MRCS part B. For the exit examination or part II WACS pass rate was 31.5% (general surgery) while it was 64% for Fellowship Royal College of Surgeons (FRCS) cumulative and 70% in the American board of surgery (ABS). Surgeon per 100 000 population was 0.69 for Nigeria compared with 11.7 and 25.6 for the UK and US respectively. In the last 35 years WACS has produced 1638 surgeons (2.8 times more than NPMCN) in surgery and NPMCN has produced 572. The frequency of examination were twice per year for both WACS and NPMCN examinations, 3 times per year for the USMLE step 3, MRCS (A & B) and Fellowship Royal College of Surgeons (FRCS) general surgery. The American Board of Surgery (ABS) is once per year for Qualifying Examination (QE) and 5 times per year for Certifying Examination (CE).

## Background

In Nigeria residency training started formally over 36 years ago with the formation of the West African College of surgeons (WACS) and the National Postgraduate medical college of Nigeria (NPMCN) [1,2]. The West African College of Surgeons grew from the Association of Surgeons of West Africa, which was founded in 1960. The decision to transform into a college was made in Accra, Ghana, in 1969. The association ceased to exist in 1973, when it transferred its assets and liabilities to the college in Benin city, Nigeria. Today residency training in surgery is well established in Nigeria in most specialties however currently areas like vascular surgery and hand surgery are not available WACS and NPMCN together with their accredited training institutions handle training, qualifying and certifying examinations. The Medical and Dental Council of Nigeria (MDCN) handles licensure for all surgical specialties. For both colleges there are three examinations to becoming a fellow of the respective college; primary, part I and part II and each is a pre-requisite for the other. Only the primary examination can be written outside a training position. In the United States (US) and United Kingdom (UK) there is currently no equivalent of the primary fellowship examination. The UK MRCS (A & B) is technically a combination of primary and part I. There is a lot of research on attraction and retention on surgeon workforce in Nigeria, but not so much on the production of surgeons, however works by Yusuf et al., Clement et al. and Yawe et al. did focus on factors that affect surgeons in training [3–7]. There is currently few if any study that considers the low surgeon production and its contribution to the low surgeon workforce in Nigeria. Suprisingly success rate in each stage of the examination varies widely between these three countries with that of Nigeria being the lowest and that of the US the highest even though surgeons trained and licensed to practice in the UK & US are less required to re-license when they go abroad compared to surgeons trained in Nigeria. These calls to question the standard setting used to assess trainee surgeons in Nigeria. West Africa and Nigeria have severe shortage of surgeon workforce, despite having surgeon producing institutions for over 36 years, mindful of other attraction and retention of surgeon factors which play their own role. I believe the examining bodies in Nigeria may be under producing surgeons. There are a number of studies and arguments that buttress this assertion and a few argument that refute this claim [3,4,8]. My paper is the first to my knowledge to review this.

**Fig. 1:**
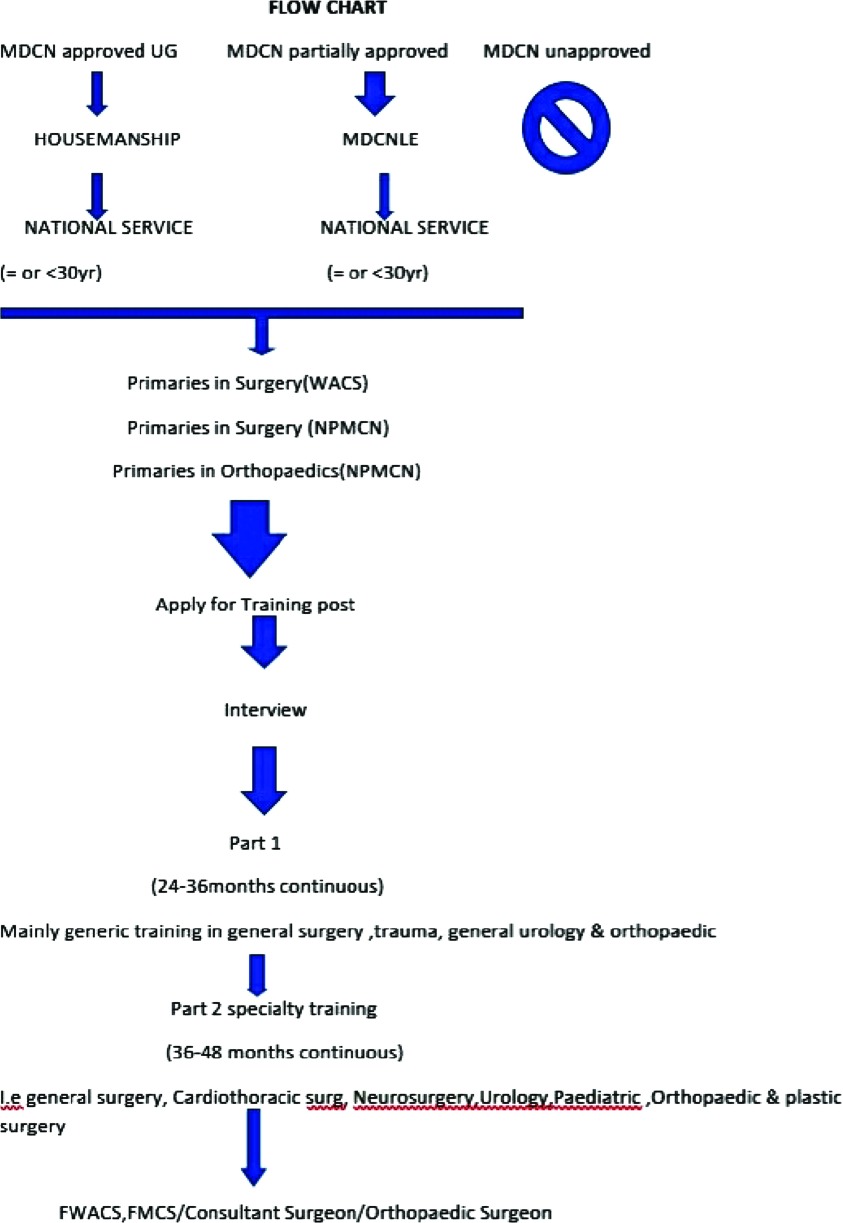
Flow chart.

Below is a flow chart depicting the pathway towards becoming a surgeon in Nigeria (Fig. 1). Regarding language of communication in training, the official language in Nigeria is English which is the language used to train and assess candidates in Nigeria for all undergraduate and postgraduate medical education. Since July 2011 the WACS faculty of Surgery has a published curriculum for all its examination available free online while the faculty of Surgery, NPMCN has a syllabus published as a book available on purchase for a fee. It is important to state that there is a national undergraduate syllabus for orthopedics which is provided to medical schools by the National Universities Commission of Nigeria and the Medical and Dental Council of Nigeria, while for postgraduate the WACS has its own orthopedic training curriculum for the part II unlike the NPMCN which has for all parts of the fellowship examination i.e. primaries, part I & II. The faculty of surgery has a faculty board of examiners headed by the faculty chairman who are appointed to a two-year term, the board are responsible for appointment of the court of examiners for each diet of examination. The criteria for the appointing examiners is not generally available to the public domain. At present there is no limit to the number of attempts a candidate can have at any of the examinations of the WACS or NPMCN, hence candidates that fail to pass an examination will in most cases apply to retake the examination after every six months until they are successful. Unfortunately, there is no data in the public domain that looks at the number of attempts by candidates for both or either examination.

The aim of this study is to draw attention to the fact the low success rate in post graduate surgical examinations may be a major contributing factor, in that it attracts a large enough pool of prospective surgeons but graduates only a small number.

## Main text

The data set was gotten from published research and official information on post graduate surgical training and related statistics from the official website of examining bodies like WACS, NPMCN, Joint Commission on Intercollegiate Examinations (JCIE), ABS, Royal College of Surgeons (RCS) and their affiliates, other bodies like National Health Service (NHS) UK, Accreditation Council of Graduate Medical Education (ACCGME), American Association of Medical Colleges (AAMC), Federation of State Medical Boards (FSMB), General Medical Council (GMC) were used as well. The following questions and answers will serve as a window to explore and discuss the information.

### What is the passrate of the west African college of surgeons' examination before?

**Fig. 2:**
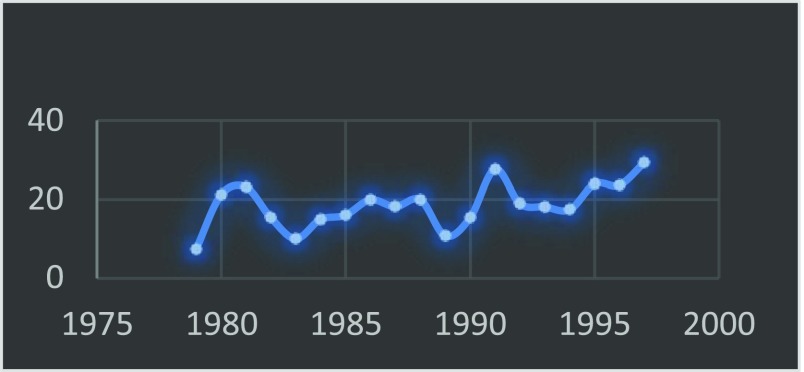
Trend of WACS passrate_Primaries.

**Fig. 3:**
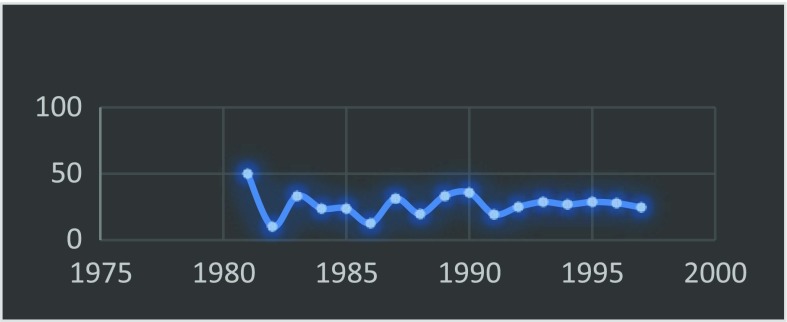
Trend of WACS passrate_Part I.

**Fig. 4:**
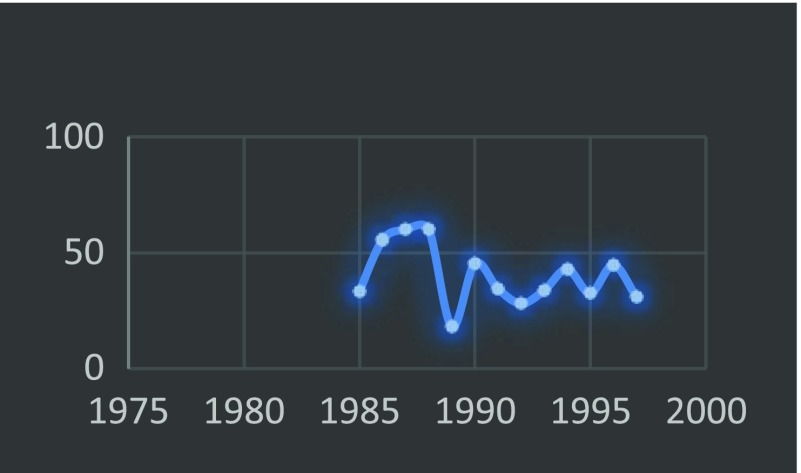
Trend of WACS passrate_Part II.

In 1999 the Journal of the American Medical Association published “Surgery in Nigeria” by Ajayi and Adebamowo which revealed that at best the trend of the outcome of the results of the WACS examination has been haphazard with a tendency more towards underproduction [4]. This is further depicted by Table 1 and Figures 2–4.

**Table 1:** Pass rate of the WACS examination from 1979 to 1997.

Year	Primary		Part I		Part II	
	Participated	Passed (%)	Participated	Passed (%)	Participated	Passed (%)
1979	27	2(7.4)	No data	No data	No data	No data
1980	33	7(21.2)	No data	No data	No data	No data
1981	56	13(23.2)	4	2(50.0)	No data	No data
1982	90	14(15.5)	10	1(10.0)	No data	No data
1983	109	11(10.1)	15	5(33.3)	No data	No data
1984	127	19(15.0)	21	5(23.8)	No data	No data
1985	118	19(16.1)	21	5(23.8)	3	1(33.3)
1986	130	26(20.0)	24	3(12.5)	9	5(55.6)
1987	197	36(18.3)	32	10(31.3)	10	6(60.0)
1988	220	44(20.0)	71	14(19.7)	5	3(60.0)
1999	204	22(10.8)	114	38(33.3)	11	2(18.2)
1990	177	28(15.5)	106	38(35.8)	11	5(45.5)
1991	184	51(27.7)	93	18(19.4)	26	9(34.6)
1992	205	39(19.0)	116	29(25.0)	39	11(28.2)
1993	209	38(18.2)	122	35(28.7)	56	19(33.9)
1994	251	44(17.5)	138	37(26.8)	58	25(43.1)
1995	196	47(24.0)	122	35(28.7)	49	16(32.7)
1996	177	42(23.7)	115	32(27.8)	69	31(44.9)
1997	218	64(29.4)	134	33(24.6)	71	22(31.0)
**Range**	–	**2–64(7–29%)**	–	**1–38(50–36%)**	–	**1–31(33–45%)**
**Median**	–	**28(5%)**	–	**18(5%)**	–	**9 (6%)**

### What is the passrate of the west African college of surgeons' examination now?

A review of recent data of the WACS examination was done to understand how candidates were performing in recent times (see the Supplemental Material for full details such as number of participating candidates from each training centre, etc.)

- A review of WACS primaries examination conducted biannually from 2015–2017 revealed passrate for the three years that ranged from 16–36% with a median of 20%.
- A review of WACS part I examination conducted biannually from 2012–2013 revealed passrate for the two years that ranged from 21–36% with a median of 29%.
- A review of WACS part I examination by regional location of training centre conducted biannually for 2016 & 2017 revealed passrate that showed marked variability by location of training centre.

For 2016 here is the summary:

I. Passrate of all training centres located in the north central ranged from 6–22%.
II. Passrate of all training centres located in the north east ranged from 0–17%.
III. Passrate of all training centres located in the north west ranged from 0–33%.
IV. Passrate of all training centres located in the south west ranged from 0–43%.
V. Passrate of all training centres located in the south east ranged from 0–67%.
VI. Passrate of all training centres located in the south south ranged from 0–22%.

For 2017 here is the summary:

I. Passrate of all training centres located in the north central ranged from 0–50% with an overall regional passrate of 16%.
II. Passrate of all training centres located in the north east ranged from 20–100% with overall regional passrate of 41%.
III. Passrate of all training centres located in the north west ranged from 0–67% with overall regional passrate of 19%.
IV. Passrate of all training centres located in the south west ranged from 17–50% with overall regional passrate of 36%.
V. Passrate of all training centres located in the south east ranged from 0–57% with overall regional passrate of 22%.
VI. Passrate of all training centres located in the south south ranged from 0–50% with overall regional passrate of 18%.

- A review of WACS part II examination by conducted biannually for 2016 & 2017 revealed passrate that showed marked variability by location of training centre.
- A review of WACS part II examination conducted biannually from 2012–2013 revealed passrate for the two years that ranged from 17–37% with a median of 32%.
- A review of WACS part II examination by regional location of training centre conducted biannually for 2016 & 2017 revealed passrate that showed marked variability by location of training centre for all specialties available in the WACS program namely cardiothoracic, general, neuro, orthopaedic, paediatric, plastic and urologic surgery (the details of these will require too much space to even summarize here so I will refer the reader to Table S.8 of the Supplemental Material).

### Does surgery attract higher quality trainees and how does passrate in surgery (including orthopaedics) compare with other faculties?

**Fig. 5:**
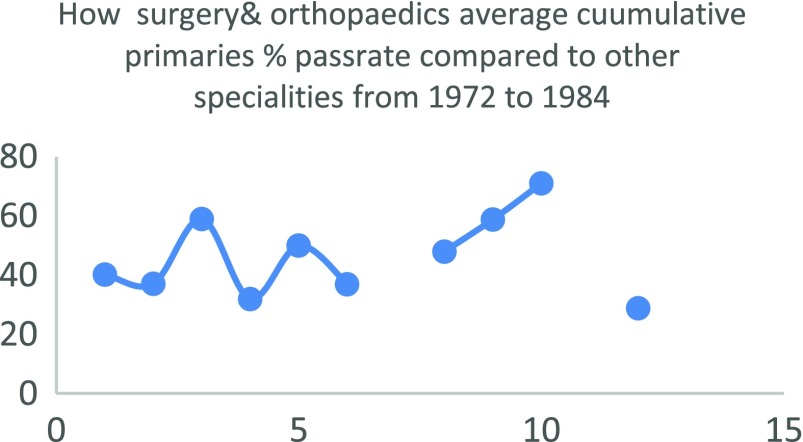
How surgery & orthopaedics average cuumulative primaries % passrate compared to other specialities from 1972 to 1984.

**Fig. 6:**
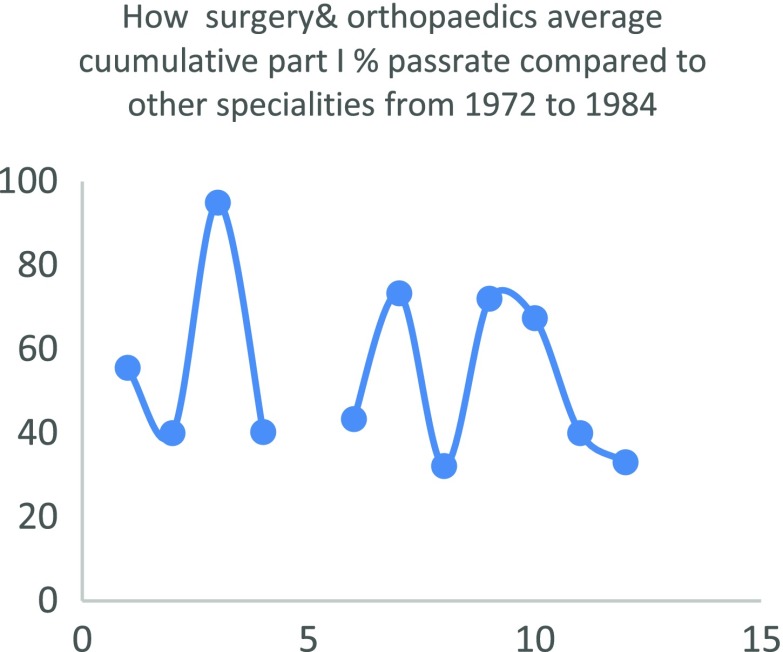
How surgery & orthopaedics average cuumulative part I % passrate compared to other specialities from 1972 to 1984.

**Fig. 7:**
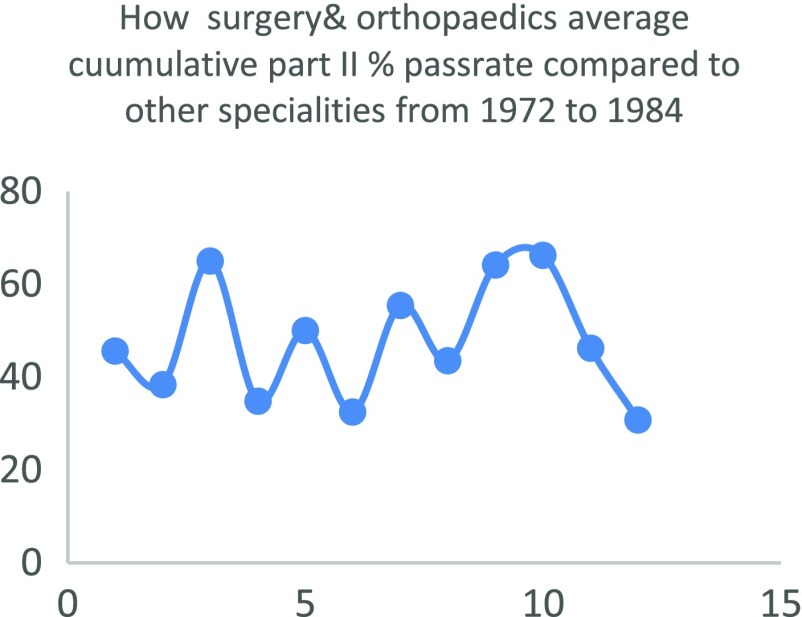
How surgery & orthopaedics average cuumulative part II % passrate compared to other specialities from 1972 to 1984.

Regarding the quality of Nigerian doctors that chose to train in surgery or orthopaedics, it is hard to pin point any objective predictor of the quality of candidates entering surgical training in the country. Nigeria does not use a single national examination to license its doctors as for instance the USMLE. Rather different medical schools are left to conduct their own examinations which is used by the medical and dental council to give the newly graduated doctor a license to practice under supervision. Therefore, there is a general lack of an objective predictor that can be used to infer that surgery takes in candidates that are any less in quality compared to other disciplines. An interesting paper by Ohwovoriole et al. in 1987 “A Review of the Fellowship Examination of the Nigerian Postgraduate Medical College (1972–84)” [9] compared the passrate of candidates in all twelve faculties of the college at that time. Surgery (including orthopaedics) lagged obstetrics and gynaecology, anaesthesia, dental surgery, public health and psychiatry in all phases of the examination. For the exit examination surgery lagged all the faculties except paediatrics and internal medicine. This is depicted in Table 2 and Figures 5–7

**Table 2:** Passrate National Postgraduate Medical College of Nigeria 1972–84.

S/N	Faculties	No of entries Primaries	No of entries Part I	No of Entries Part II	Total
1	Anaesthesia	24/59(40.1%)	10/18(55.6%)	2/2(100%)	36/79(45.6%)
2	Dental surgery	26/70(37.1%)	6/15(40%)	1/1(100%)	33/86(38.4%)
3	General Medical Practice	62/105(59%)	21/22(95%)	No data	83/127(65%)
4	Obstetrics & Gynaecology	272/847(32.3%)	97/216(40.3%)	34/66(54.5%)	393/1129(34.8%)
5	Ortorhinolaryngology	1/2(50%)	No data	No data	1/2 (50%)
6	Paediatrics	153/415(36.9%)	61/250(43.3%)	13/30(43.3%)	226/695(32.5%)
7	Pathology	No data	29/57(73.3%)	11/15(73.3%)	40/72(55.5%)
8	Internal medicine	231/481(48%)	92/281(32.1%)	36/57(30.6%)	359/825(43.5%)
9	Psychiatry	30/51(58.8%)	18/25(72%)	4/5(80%)	52/81(64.2%)
10	Public health	44/62(71%)	31/46(67.4%)	9/15(60%)	84/123(66.3%)
11	Radiology	No data	12/30 (14%)	4/6(66.7%)	18/39(46.2%)
12	Surgery	208/730(28.5%)	87/203(33.1%)	27/53(50.9%)	322/1046(30.8%)

### What are the reasons for failure by candidates?

Some WACS examiners like Ajao and Ugwu made some empirical association which in their opinion is which responsible for failure in majority of cases; poor preparation, error in case presentation, incomplete answers to questions, poor knowledge of basic medical sciences, poor knowledge and practice of evidence based medicine and critical appraisal skills [6].

For each of the reasons a number of solution exist which is backed by some level of evidence; better preparation has been shown by researchers to be a significant predictor of candidates passing surgery and orthopaedics examinations [10–12] training on examination process which relates to errors in long and short case is supported by studies by Paula et al., Scrimgeour and Chipp and colleagues [10,13–15]. Revision with model answers will prevent candidates making the error of incomplete answers to questions while better preparation with structured revision in basic medical sciences will prevent candidates from gross deficiency in the knowledge of basic medical science relevant to surgery and orthopaedic are supported by the fact that higher scores in preceding examination is generally associated with a higher chance of passing the subsequent examination on first attempt [11–13]. Providing free or subsidized access to journals will help candidates demonstrate good knowledge of evidence based practice was associated with higher success rate by orthopaedic surgical trainees [11].

### How does local surgery training factors including training slots and recruitment compare with other countries?

Tables 3–5 try to answer the above question.

**Table 3:** Demographics of different stage of post graduate surgical exams [25–32].

Country	Examining bodies	Total accredited training programs	Approx. Yearly residency training slots	Aggregated Pass rate Primary /equivalent Frequency of exams per year	Aggregated Average pass rate Part I/equivalent 2012–2013 Frequency of exams per year	Aggregated average pass rate Part II surgery or exit 2012–2013 Frequency of exams per year	Country population
Nigeria [9]	WACS^b^	None 46(WACS) 52(NPMCN)	572^a^	<40% 2×	28.8% 2×	31.5%(general surgery) 2×	180M
UK	Royal Colleges of surgery	LETB [23] 13 Deaneries 3 99	646	None	MRCS A 36.6% [24] MRCS B 57.5% [24] 3×	FRCS(general surgery) 51% 3×	65M
US	USMLE (FSMB)s American board of surgery	1 964	4225	USMLE[12] step 3 98% 3×	None	ABS (general surgery) QE 80% 1× CE 76% 5×	324M

a The figure represented the total accredited training slots by the WACS, there is no available data of the total yearly intake of resident, but there has being very poor uptake of residents by training institutions in the last three years in all parts of the country according to opinion sampled from surgery program directors.

b I decided to use the WACS statistics because it is the most popular examination and it has a higher pass rate, the statistics presented are not without flaws as WACS till date does not publish analysis of pass and failure rate of its examinations to the public domain.

**Table 4:** Surgical workforce (surgeons) [29–32].

Country	Total surgeons as of	Surgeon per 100000
Nigeria	1246(2014)	0.69
UK	7540(2015)	11.6
USA	83416(2015)	25.7

**Table 5:** Surgeons produced in Nigeria in the last 35 years  [25,26].

Surgery subspecialties	WACS	NPMCN
Surgery (All except below)	No data	232
ENT	No data	74
Dental surgery	No data	144
Orthopaedics	No data	122
Total	1635	572

Information on the volume of surgeons Nigeria as of 2014 according to MDCN was gotten from a journal article published by African Centre for Global Health And Social Transformation (ACHEST) from which the surgeon per 100 000 was calculated [16]. While the volume of surgeons in the UK was gotten from RCS and that of the US from AAMC and the surgeon per 100 000 calculated and results displayed as follows [1,17].

The WACS information did not state which fellows where by examination while NPMCN's list of dissertations was used and hence it is likely to be purely by examination. A sizable number of surgeons have dual fellowship however it was not possible to disaggregate mainly due to lack of access to the data.

### How surgeons (including orthopaedic surgeons) dropout rate relates to surgical workforce?

There are no data on the general or disaggregated dropout rate for surgical or orthopaedic trainees in Nigeria. Therefore, it is it is difficult to objectively answer the question of how many trainees leave the training due to failure to pass an examination after one or more attempts. According to the guidelines of the WACS and NPMCN, training in orthopaedics and surgery takes about 5.5 to 6 years. In Nigeria trainees are given a period of six year with an opportunity for a year extension under certain strict circumstances by training institutions to finish or withdraw from the training. Again, there is no data on the number of surgeons and orthopaedic surgeons leaving the country after completion of training but a recent publication in 2017 reveals the information below. See Table 6 for more information.

**Table 6:** Statistics on Nigerian Doctors.

Doctors	Percentage
Home	48%
Abroad	52%
Seeking job abroad	88%
Source	Nigerian Medical Association & www.eureka.com.ng

## Discussion

As regards frequency of examination, WACS and NPMCN holds all stages of its examination twice per year, while USMLE step 3, the MRCS (A and B) and FRCS (all specialty) are held thrice per year [1,2,16]. The general surgery ABS QE is held once yearly while the ABS CE is held 5 times per year [18]. Regarding frequency of assessing candidates UK examining bodies clearly examine candidates more frequently and the General ABS CE is also clearly more frequent.

The above results buttress the popular believe that surgical training examining institutions and programs are better funded and given more attention in the UK and US compared to Nigeria, it also shows that the standard setting used by these foreign bodies may be better. This is sad considering the high unmet need for surgical care in Nigeria and Africa. Structurally there are fewer accredited training programs, fewer training slots yearly for doctors seeking to train in surgery. In Nigeria the examining bodies combine the dual role of examining and accrediting institutions for post graduate surgical training [1,2]. In the UK, examining role is handled by the royal colleges, while the GMC handles accreditation of training programs [16,19]. Similarly, in the US the American board of surgery handles examination of trainees while ACGME handles accreditation of training programs [18,20]. This may suggest that the roles may be best handled by two different bodies, however further research needs to be done to better clarify this. The pass rate which is a very important factor for all stakeholders; trainees, training programs as well as examining and accrediting bodies was analyzed for the three postgraduate surgical examinations i.e. primary, part I and II and compared to its equivalent where applicable in the US and UK. The primary examination which is comparable with the step 3 USMLE which has a aggregated passrate of 98%, one article written by WACS examiners states an average pass rate of less than 40% [3,21]. This exam which serves as the entry point to postgraduate surgical training in Nigeria with such a low pass rate may be making surgery less attractive to medical students and fresh medical graduates; however more research needs to be done to shed more light to this statement. The part I (written and clinical) with its counterparts the MRCS part A and B are in-training examination, hence a lot more factors have being shown to influence the ability of candidates' pass rate by several studies [3,6]. Again in this examination the average aggregated pass rate for WACS is 28.8% while that of the MRCS A is 36.6% and MRCS B is 57.5% again Nigeria falling behind [3,16]. For the exit or part II the average general surgery pass rate was used, because it is more feasible to compare pass rate in general surgery for all three countries. That of WACS was 31.5% and of its counterparts was 51% for FRCS general surgery and 76% QE and 80% CE for ABS general surgery. Again Nigeria falls behind [3,17,18]. The frequency to which candidate where being examined yearly was less for examining bodies in Nigeria compared to the UK and US. The surgeon per 100 000 population of the year just following average of aggregated passrate was 0.69 for Nigeria, 11.6 for the UK and 25.7 for the US further buttressing the link of low surgeon workforce and success in postgraduate surgical training. The results also showed that when the two main post graduate surgical colleges were compared WACS has produced 2.8 times more surgeons in the last 35 years compared to NPMCN. It is important to note that however a good number of surgeons hold dual fellowship. The reason for this wide disparity is most likely associated with the lower pass rate of NPMCN examinations as well as a lesser volume of candidates who sit for their exams yearly. Further insight into this difference requires more research.

### The role of overseas examiners and assessors in resetting the standard of the examinations

It may be helpful to get assessors and examiners from some of the oldest organized surgical colleges from different continents of the world like the royal colleges from Europe, the American board from North America and the College of Medicine of South Africa to help in resetting the standard of the examinations. The various college of surgeons have a long history of helping in such standard setting around the world most especially the royal colleges.

## Conclusion

Based on the findings above amongst other factors the low production of surgeons in Nigeria is likely a significant contributor to the low volume of surgeons in the country. A review of the way surgeons in training are trained and assessed, especially the standard setting used needs to be reviewed. Luckily the federal government just commissioned a committee to review residency program in Nigeria [22]. An urgent review of the standard setting for postgraduate surgical examinations of the West African college of surgeons and the National postgraduate college may be required to reduce the attrition rate of surgical residents in the country.

## Conflict of interest

No conflict of interest except that the author is a trainee surgeon.

## Supplementary Material

Table S1 WACS primaries.
Table S2–S4 WACS part I.
Table S5–S8 WACS part II. The Supplementary Material is available at https://www.sicot-j.org/10.1051/sicotj/2018008/olm.

## List of abbreviations

ABS: American Board of Surgery
ABS CE: American Board of Surgery Certifying Examination
ABS QE: American Board of Surgery Qualifying Examination
ACHEST: African Centre for Global Health and Social Transformation
ACCGME: Accreditation Council for Graduate Medical Education
AAMC: Association of American Medical Colleges
FRCS: Fellow Royal College of Surgeons
FSMB: Federation of State Medical Board
GMC: General Medical Council
JCIE: Joint Commission on Intercollegiate Examination
MDCN: Medical and Dental Council of Nigeria
MRCS: Member Royal College of Surgeons
NHS: National Health Service UK
NPMCN: National Postgraduate Medical College of Nigeria
RCS: Royal College of Surgeons
UK: United Kingdom
US: United States of America
USMLE: United States Medical Licensing Examination
WACS: West African College of Surgeons
JUTH: Jos University Teaching Hospital
UITH: University of Ilorin Teaching Hospital
DASH: Dalhatu Araf Specialist Hospital
BSUTH: Benue State University Teaching Hospital
UATH: University of Abuja Teaching Hospital
NHA: National Hospital Abuja
FMCM: Federal Medical Centre Makurdi
FMCL: Federal Medical Centre Lokoja
FMCB: Federal Medical Centre Bida
FCDA: Federal Capital Development Authority
ATBUTH: Abubakar Tafawa Balewa University Teaching Hospital
UMTH: University of Maiduguri Teaching Hospital
FTHG: Federal Teaching Hospital Gombe
FMCYo: Federal Medical Centre Yola
FMCN: Federal Medical Centre Nguru
ABUTH: Ahmadu Bello University Teaching Hospital
AKTH: Aminu Kano Teaching Hospital
UDUTH: Usman Danfodio Teaching Hospital
NOHK: National Orthopaedic Hospital Kano
FMCK: Federal Medical Centre Katsina
UCH: University College Hospital
LUTH: Lagos University Teaching Hospital
OAUTHC: Obafemi Awolowo University Teaching Hospital Complex
LASUTH: Lagos State University Teaching Hospital
LAUTECH: Ladoke Akintola University of Technology
EkitiSUTH: Ekiti State University Teaching Hospital
OOUTH: Olabisi Onabanjo University Teaching Hospital
NOHI: National Orthopaedic Hospital Igbobi
FMCI: Ido Ekiti
FMCO: Federal Medical Centre Owo
OTCS: Ondo Trauma Centre
UNTH: University of Nigeria Teaching Hospital
NAUTH: Nnamdi Azikiwe University Teaching Hospital
FETHA: Federal Teaching Hospital Abakiliki
ABSUTH: Abia State University Teaching Hospital
ESUTH: Enugu State University Teaching Hospital
IMSUTH: Imo State University Teaching Hospital Abuja
NOHE: National Orthopaedic Hospital Enugu
FMCOw: Federal Medical Centre Owerri
FMCU: Federal Medical Umuahia
UBTH: University of Benin Teaching Hospital
UCTH: University of Calabar Teaching Hospital
UPTH: University of Port Harcourt Teaching Hospital
UUTH: University of Uyo Teaching Hospital
DELSUTH: Delta State University Teaching Hospital
ISTH: Irrua Specialist Teaching Hospital
FMCA: Federal Medical Centre Asaba
FCMYe: Federal Medical Centre Yenagoa
