# Supplementary material for: Low Pass-rate in postgraduate surgical examination in Nigeria and its contribution to the low surgeon workforce in the country; a review article
Source: SICOT J. 2018 Sep 3;4:36. doi: 10.1051/sicotj/2018008 (PMC6120751; doi:10.1051/sicotj/2018008)
Supplement: Supplementary file 1 — Table S1 WACS primaries. Table S2–S4 WACS part I. Table S5–S8 WACS part II. [file sicotj-4-36-s1.pdf]

What is the passrate of the west African college of surgeons' examination now?

WACS Primaries

Table 1;

**APRIL**				**October**			
	Eligible	Successful	Pass rate		Eligible	Successful	Pass rate
**2015**	368	136	36%	**2015**	No data	No data	No data
**2016**	329	62	19%	**2016**	327	62	19%
**2017**	193	30	16%	**2017**	168	41	24%
**Range**			**36-16**				**24-19**
**Median**			**19**				**21.5**

WACS Part I

Table 2;

**April**				**October**			
Year	Eligible	Successful	Pass rate	year	Eligible	Successful	Pass rate
**2012**	197	59	30%	**2012**	178	55	31%
**2013**	177	37	21%	**2013**	168	56	33%
**Range**			**30-21**				**33-31**
**Median**			**25.5**				**32**

Table 3

**April**					**Oct**				
Year	Centre	Eligible cand	Successful cand	Pass rate	Year	centre	Eligible cand	Successful cand	Passrate
**2016**					**2016**				
	**North central**					**North central**			
	JUTH	16	No data	No data		JUTH	15	1	6%
	UITH	15	No data	No data		UITH	9	2	22%
	DASH					DASH	0	0	0%
	BSUTH					BSUTH	1	0	0%
	UATH	19	No data	No data		UATH	14	4	29%
	NHA	10	No data	No data		NHA	6	0	0%
	FMCM	3	No data	No data		FMCM	4	0	0%
	FMCL	3	No data	No data		FMCL	3	0	0%
	FMCB	3	No data	No data		FMCB	2	0	0%
	FCDA					FCDA	0	0	0%
	**NORTH EAST**					**NORTH EAST**			
	ATBUTH	2	No data	No data		ATBUTH	0	0	0%
	UMTH	4	No data	No data		UMTH	4	0	0%
	FTHG	1	No data	No data		FTHG	6	1	17%
	FMCYo					FMCYo	0	0	0%
	FMCN					FMCN	0	0	0%
	**NORTH**					**NORTH**			

	**WEST**					**WEST**			
	ABUTH	10	No data	No data		ABUTH	7	0	0%
	AKTH	5	No data	No data		AKTH	4	1	25%
	UDUTH	5	No data	No data		UDUTH	4	1	25%
	NOHK					NOHK	3	1	33%
	FMCK					FMCK	2	0	0%
	**SOUTH WEST**					**SOUTH WEST**			
	UCH	6	No data	No data		UCH	7	3	43%
	LUTH	6	No data	No data		LUTH	2	0	0%
	OAUTH	3	No data	No data		OAUTH	7	5	71%
	LASUTH	6	No data	No data		LASUTH	10	1	10%
	LAUTECH	5	No data	No data		LAUTECH	4	1	25%
	EkitiSUTH					EkitiSUTH	0	0	0%
	OOUTH	3	No data	No data		OOUTH	4	1	25%
	NOHI	3	No data	No data		NOHI	0	0	0%
	FMCI					FMCI	0	0	0%
	FMCO	3	No data	No data		FMC0	4	2	50%
	OTCS					OTCS		0	0%
	**SOUTH EAST**					**SOUTH EAST**			
	UNTH	6	No data	No data		UNTH	6	4	67%
	NAUTH	8	No data	No data		NAUTH	5	4	80%
	FETHA	13	No data	No data		FETHA	12	6	50%
	ABSUTH	3	No data	No data		ABSUTH	1	0	0%
	ESUTH	5	No data	No data		ESUTH	3	0	0%
	IMSUTH	4	No data	No data		IMSUTH	4	1	25%
	NOHE	14	No data	No data		NOHE	4	3	75%
	FMCOw	5	No data	No data		FMCOw	10	0	0%
	FMCU	3	No data	No data		FMCU	5	2	40%
	**SOUTH SOUTH**					**SOUTH SOUTH**			
	UBTH	16	No data			UBTH	9	2	22%
	UCTH	4	No data			UCTH	4	0	0%
	UPTH	12	No data			UPTH	11	1	9%
	UUTH	8	No data			UUTH	6	0	0%
	DELSUTH	4	No data			DELSUTH	3	1	33%
	ISTH	6	No data			ISTH	2	1	50%
	FMCA	4	No data			FMCA	5	0	0%
	FCMYe	3	No data			FMCYe	4	0	0%
**Range**									**80-0**
**Median**									**0.0**

Table 4

**April**					**Oct**				
Year	centre	Eligible cand	Successful cand	Pass rate	Year	centre	Eligible cand	Successful cand	Passrate
**2017**					**2017**				
	**North central**	41	7	13%		**North central**	49	9	18%
	JUTH	10	4	40%		JUTH	11	2	18%
	UITH	3	0			UITH	3	1	33%
	DASH	2	1	50%		DASH			
	BSUTH	1	0			BSUTH	4	0	0%
	UATH	10	1	11%		UATH	5	0	0%

	NHA	5	1	20%		NHA	15	5	33%
	FMCM	3	0	0%		FMCM	3	0	0%
	FMCL	7	0	0%		FMCL	6	1	16%
	FMCB	2	0	0%		FMCB	2	0	0%
	FCDA					FCDA			
	**NORTH EAST**	13	4	31%		**NORTH EAST**	8	4	50%
	ATBUTH	1	1	100%		ATBUTH	2	1	50%
	UMTH	7	2	29%		UMTH	3	2	66%
	FTHG	5	1	20%		FTHG	3	1	33%
	FMCYo					FMCYo			
	FMCN					FMCN			
	**NORTH WEST**	16	3	19%		**NORTH WEST**	**21**	4	19%
	ABUTH	4	1	25%		ABUTH	3	0	0%
	AKTH	3	0	0%		AKTH	6	2	33%
	UDUTH	4	0			UDUTH	6	2	33%
	NOHK	1	0			NOHK	3	0	0%
	FMCK	3	2	67 %		FMCK	3	0	0%
	**SOUTH WEST**	48	19	40%		**SOUTH WEST**	45	14	31%
	UCH	25	12	48%		UCH	23	10	43%
	LUTH	2	1	50%		LUTH	3	1	33%
	OAUTH	5	2	40%		OAUTH	4	0	0%
	LASUTH	6	1	17%		LASUTH	4	0	0%
	LAUTECH	4	2	50%		LAUTECH	3	0	0%
	EkitiSUTH					EkitiSUTH			
	OOUTH	1	0			OOUTH			
	NOHI	3	0			NOHI	7	3	43%
	FMCI					FMCI			
	FMCO	2	1	50%		FMC0	1	0	0%
	OTCS					OTCS			
	**SOUTH EAST**	45	10	22%		**SOUTH EAST**	49	11	22%
	UNTH	2	0	0%		UNTH	5	1	20%
	NAUTH	1	1	100%		NAUTH			
	FETHA	18	7	39%		FETHA	12	2	16%
	ABSUTH	1	0	0%		ABSUTH	1	0	0%
	ESUTH	3	0	0%		ESUTH	6	1	16%
	IMSUTH	5	0	0%		IMSUTH	4	0	0%
	NOHE	5	0	0%		NOHE	8	1	13%
	FMCOw	5	0	0%		FMCOw	7	4	57%
	FMCU	5	2	40%		FMCU	6	2	33%
	**SOUTH SOUTH**	42	6	14%		**SOUTH SOUTH**	39	8	21%
	UBTH	7	0	0%		UBTH	6	2	33%
	UCTH	4	0	0%		UCTH	4	0	0%
	UPTH	9	1	11%		UPTH	5	2	40%
	UUTH	4	0	0%		UUTH	4	1	25%
	DELSUTH	6	2	33%		DELSUTH	5	1	20%
	ISTH	4	2	50%		ISTH	5	0	0%
	FMCA	7	1	14%		FMCA	6	2	33%
	FCMYe	1	0	0%		FMCYe	4	0	0%
**Range**				**100-0**					**66-0**
**Median**				**11.0**					**17.0**

WACS part II

Table 5

APRIL				October			
Year	Eligible	Successful	Pass rate	Year	Eligible	Successful	Pass rate
**2012**	25	9	36%	**2012**	19	7	37%
**2013**	18	3	17%	**2013**	22	8	36%
**Range**			**36-17**				**37-36**
**Median**			**26.5**				**36.5**

Table 6

Year	centre	Eligible candidates	Successful candidates	Pass rate
**2014 APRIL**				
	**North central**		No data	No data
	JUTH	2	No data	No data
	UITH	1	No data	No data
	DASH		No data	No data
	BSUTH		No data	No data
	UATH	1	No data	No data
	NHA	2	No data	No data
	FMCM		No data	No data
	FMCL		No data	No data
	FMCB	2	No data	No data
	FCDA			
	**NORTH EAST**		No data	No data
	ATBUTH		No data	No data
	UMTH	1	No data	No data
	FTHG			
	FMCYo			
	FMCN			
	**NORTH WEST**			
	ABUTH	3	No data	No data
	AKTH	3	No data	No data
	UDUTH	2	No data	No data
	NOHK	2	No data	No data
	FMCK			
	**SOUTH WEST**		No data	No data
	UCH	4	No data	No data
	LUTH	5	No data	No data
	OAUTH	5	No data	No data
	LASUTH	5	No data	No data
	LAUTECH	1	No data	No data
	EkitiSUTH			
	OOUTH			
	NOHI	2	No data	No data
	FMCI			
	FMCO	1	No data	No data
	OTCS			

	**SOUTH EAST**			
	UNTH	1	No data	No data
	NAUTH	4	No data	No data
	FETHA			
	ABSUTH			
	ESUTH			
	IMSUTH	1	No data	No data
	NOHE	4	No data	No data
	FMCOw			
	FMCU	1	No data	No data
	**SOUTH SOUTH**			
	UBTH	5	No data	No data
	UCTH	1	No data	No data
	UPTH	3	No data	No data
	UUTH			
	DELSUTH			
	ISTH			
	FMCA			
	FCMYe			
**Range**				
**Median**				

Table 7

**April**										**Oct**								
Year	cent	**EC SC PR**	**CTS**	GS	NS	OS	PS	PLs	UL	cent	**EC SC PR**	C T S	GS	NS	OS	PS	PLs	UL
**2016**										**2016**								
	**North central**									**North Central**								
	JUTH									JUTH			1<br>1<br>100%			1<br>0<br>0%		1<br>1<br>100%
	UITH									UITH					1<br>1<br>100%	2<br>1<br>50%		
	UATH									UATH								
	NHA									NHA			1<br>1<br>100%	1<br>1<br>100%				
	FMCM									FMCM							1<br>0<br>0%	
	FMCL									FMCL								
	**NORTH EAST**									**NORTH EAST**								
	UMTH									UMTH					2<br>0<br>0%		1<br>0<br>0%	
	FTHG									FTHG								
	**NORTH WEST**									**NORTH WEST**								
	ABUTH									ABUTH					1<br>0<br>0%	1<br>0<br>0%		
	AKTH									AKTH			1<br>0<br>0%				1<br>0<br>0%	1<br>0<br>0%
	UDUTH									UDUTH			1<br>0<br>0%					
	NOHK									NOHK					2<br>0<br>0%			
	**SOUTH WEST**									**SOUTH WEST**								
	UCH									UCH				2	3			

														2<br>100%	1<br>33%			
	LUTH									LUTH				2<br>0<br>0%	2<br>0<br>0%			1<br>1<br>100%
	OAUTH									OAUTH			2<br>1<br>50%	1<br>0<br>0%	3<br>1<br>33%	2<br>0<br>0%		
	LASUTH									LASUTH					2<br>1<br>50%		2<br>1<br>50%	
	LAUTEC<br>H									LAUTECH					1<br>0<br>0%			
	NOHI									NOHI					1<br>1<br>100%			
	FMCO									FMCO					1<br>0<br>0%			
	**SOUTH EAST**									**SOUTH EAST**								
	UNTH									UNTH						1<br>0<br>0%		
	NAUTH									NAUTH				1<br>0<br>0%				4<br>2<br>50%
	FETHA									FETHA								
	ABSUTH									ABSUTH								
	ESUTH									ESUTH								
	IMSUTH									IMSUTH								
	NOHE									NOHE					1<br>1<br>100%			
	FMCOw									FMCOw								
	**SOUTH SOUTH**									**SOUTH SOUTH**								
	UBTH									UBTH			1<br>0<br>0%	1<br>1<br>100%	3<br>0<br>0%			1<br>0<br>0%
	UCTH									UCTH			1<br>0<br>0%					
	UPTH									UPTH			1<br>0<br>0%	1<br>1<br>100%				2<br>0<br>0%
	UUTH									UUTH								
	ISTH									ISTH								
**Range**													**100-0**	**100-0**	**100-0**	**50-0**	**50-0**	**100-0**
**Median**													**0.0**	**100**	**0.0**	**0.0**	**0.0**	**25**

Table 8

**April**										**Octobe<br>r**								
Year	Centre	**E<br>C<br>S<br>C<br>P<br>R**	**CTS**<br>EC<br>SC<br>PR	GS	NS	OS	PS	PLS	UL	Centre	**EC<br>SC<br>PR**	CTS	GS	NS	OS	PS	PLS	UL
**2017**										**2017**								
	**North central**									**North Central**								
	JUTH					1<br>0<br>0%	1<br>0<br>0%		1<br>0<br>0%	JUTH					1<br>0<br>0%	1<br>0<br>0%		2<br>0<br>0%
	UITH						1<br>0<br>0%			UITH			1<br>0<br>0%	1<br>0<br>0%		2<br>0<br>0%		
	UATH								2<br>1<br>50%	UATH			1<br>0<br>0%			1<br>0<br>0%		
	NHA									NHA								
	FMCM									FMCM								
	FMCL									FMCL								
	**NORTH EAST**									**NORTH EAST**								
	UMTH					2		1		UMTH					1		1	

						0<br>0%		0<br>0%							1<br>100%		0<br>0%	
	FTHG			1<br>0<br>0%						FTHG								
	**NORTH WEST**									**NORTH WEST**								
	ABUTH					2<br>0<br>0%	1<br>1<br>100%			ABUTH			1<br>0<br>0%		2<br>0<br>0%			1<br>1<br>100%
	AKTH								1<br>0<br>0%	AKTH			2<br>0<br>0%					1<br>0<br>0%
	UDUTH									UDUTH				1<br>0<br>0%				
	NOHK					1<br>0<br>0%				NOHK					5<br>0<br>0%			
	**SOUTH WEST**									**SOUTH WEST**								
	UCH					4<br>4<br>100<br>%				UCH		1<br>0<br>0%	1<br>0<br>0%					1<br>0<br>0%
	LUTH		1<br>0<br>0%		4<br>1<br>25%	2<br>1<br>50%				LUTH		1<br>0<br>0%	1<br>0<br>0%	3<br>1<br>33%	1<br>0<br>0%			
	OAUTH			2<br>1<br>50%	1<br>1<br>100%	3<br>2<br>67%	1<br>0<br>0%		2<br>1<br>50%	OAUTH			2<br>0<br>0%	1<br>0<br>0%	1<br>1<br>100%	2<br>0<br>0%		1<br>1<br>100%
	LASUTH					1<br>0<br>0%		1<br>1<br>100%		LASUT<br>H					2<br>1<br>50%			
	LAUTECH					1<br>0<br>0%				LAUTEC<br>H					1<br>0<br>0%			
	NOHI					2<br>1<br>50%		1<br>1<br>100%		NOHI					3<br>1<br>33%		1<br>0<br>0%	
	FMCO					3<br>0<br>0%									1<br>0<br>0%			1<br>1<br>100%
	**SOUTH EAST**									**SOUTH EAST**								
	UNTH			1<br>0<br>0%	1<br>0<br>0%	1<br>0<br>0%	1<br>0<br>0%			UNTH			1<br>1<br>100%	1<br>1<br>100%	1<br>1<br>100%	2<br>1<br>50%		
	NAUTH			3<br>0<br>0%	1<br>0<br>0%				2<br>1<br>50%	NAUTH			3<br>1<br>33%	1<br>1<br>100%	1<br>0<br>0%			1<br>0<br>0%
	FETHA			1<br>0<br>0%						FETHA			2<br>0<br>0%			1<br>0<br>0%		
	NOHE					5<br>1<br>20%				NOHE					5<br>2<br>40%			
	FMCOw						1<br>0<br>0%			FMCO<br>w								
	**SOUTH SOUTH**									**SOUTH SOUTH**								
	UBTH			2<br>0<br>0%		2<br>1<br>50%	1<br>0<br>0%		1<br>0<br>0%	UBTH			3<br>0<br>0%		2<br>0<br>0%	2<br>1<br>50%		
	UCTH		1<br>0<br>0%	1<br>0<br>0%						UCTH			1<br>0<br>0%					
	UPTH					1<br>0<br>0%			1<br>0<br>0%	UPTH			3<br>0<br>0%		2<br>0<br>0%			1<br>0<br>0%
	UUTH							1<br>0<br>0%		UUTH								
	ISTH									ISTH								1<br>0<br>0%
**Range**			**0-0**	**50-0**	**100-0**	**100<br>-0**	**100-0**	**100-0**	**50-<br>0**			**0-0**	**100-0**	**100-0**	**100-0**	**50-0**	**0-0**	**100-0**
**Median**			**0**	**0**	**12-5**	**0.**	**0**	**50**				**0.**	**0**	**16.6**	**0**	**0**	**0**	**0**
